# Development of pulmonary arterial hypertension in mice over-expressing S100A4/Mts1 is specific to females

**DOI:** 10.1186/1465-9921-12-159

**Published:** 2011-12-20

**Authors:** Yvonne Dempsie, Margaret Nilsen, Kevin White, Kirsty M Mair, Lynn Loughlin, Noona Ambartsumian, Marlene Rabinovitch, Margaret R MacLean

**Affiliations:** 1 College of Medical, Veterinary and Life Sciences, Institute of Cardiovascular and Medical Sciences, University of Glasgow, Glasgow, UK; 2 Department of Tumor Microenvironment and Metastases, Institute for Cancer Biology, Danish Cancer Society, Copenhagen, Denmark; 3 Department of Pediatrics, Stanford University School of Medicine, Stanford, California, USA

## Abstract

**Background:**

Idiopathic and familial forms of pulmonary arterial hypertension (PAH) occur more frequently in women than men. However, the reason for this remains unknown. Both the calcium binding protein S100A4/Mts1 (Mts1) and its endogenous receptor (receptor for advanced glycosylation end products; RAGE) have been implicated in the development of PAH. We wished to investigate if the Mts1/RAGE pathway may play a role in the gender bias associated with PAH.

**Methods:**

We investigated the effects of gender on development of PAH in mice over-expressing Mts1 (Mts1+ mice) via measurement of pulmonary arterial remodeling, systolic right ventricular pressure (sRVP) and right ventricular hypertrophy (RVH). Gender differences in pulmonary arterial Mts1 and RAGE expression were assessed by qRT-PCR and immunohistochemistry. Western blotting and cell counts were used to investigate interactions between 17β-estradiol, Mts1 and RAGE on proliferation of human pulmonary artery smooth muscle cells (hPASMCs). Statistical analysis was by one-way analysis of variance with Dunnetts post test or two-way analysis of variance with Bonferronis post test, as appropriate.

**Results:**

Female Mts1+ mice developed increased sRVP and pulmonary vascular remodeling, whereas male Mts1+ mice remained unaffected. The development of plexiform-like lesions in Mts1+ mice was specific to females. These lesions stained positive for both Mts1 and RAGE in the endothelial and adventitial layers. Expression of pulmonary arterial Mts1 was greater in female than male Mts1+ mice, and was localised to the medial and adventitial layers in non plexiform-like pulmonary arteries. RAGE gene expression and immunoreactivity were similar between male and female Mts1+ mice and RAGE staining was localised to the endothelial layer in non plexiform-like pulmonary arteries adjacent to airways. In non-plexiform like pulmonary arteries not associated with airways RAGE staining was present in the medial and adventitial layers. Physiological concentrations of 17β-estradiol increased Mts1 expression in hPASMCs. 17β-estradiol-induced hPASMC proliferation was inhibited by soluble RAGE, which antagonises the membrane bound form of RAGE.

**Conclusions:**

Mts1 over-expression combined with female gender is permissive to the development of experimental PAH in mice. Up-regulation of Mts1 and subsequent activation of RAGE may contribute to 17β-estradiol-induced proliferation of hPASMCs.

## Background

Pulmonary arterial hypertension (PAH) is a progressive disease associated with increased constriction and remodeling of the pulmonary vasculature. This leads to right heart failure and mean survival time in PAH patients is typically less than 3 years. Both idiopathic PAH and familial PAH occur more frequently in women than men (>2:1) [1-3]. Despite this, the underlying reason for the increased prevalence in women remains unclear. This has been difficult to investigate in animal models of PAH as male rats are more prone than females to hypoxia-induced PAH and paradoxically, estrogens protect against monocrotaline-induced PAH [4,5].

S100A4/Mts1 (Mts1) is a member of the S100 family of small calcium binding proteins. On interaction with calcium, a conformational change exposes a hydrophobic domain which can interact with and regulate the function of target proteins. Mts1 has been implicated in the pathogenesis of both human and experimental PAH. For example, Mts1 is up-regulated in the neointima and adventitia of occlusive and early plexiform lesions in PAH patients [6]. Moreover, mice over-expressing Mts1 (Mts1+ mice) develop increased right ventricular systolic pressure (sRVP) [7] and a subset of these mice develop pulmonary arterial remodeling similar to human plexogenic lesions [6]. These lesions are associated with a heightened activity of elastase and pulmonary artery elastin degradation [8,9]. Mts1 is synthesized and released from human pulmonary artery smooth muscle cells (hPASMCs) in response to serotonin. It can then act in an autocrine fashion to mediate the proliferation and migration of hPASMCs via activation of the receptor for advanced glycosylation end products (RAGE) [10]. The primary defect of familial PAH is a mutation in the gene encoding bone morphogenetic protein receptor type 2 (BMPRII) [11] and this is present in at least 70% of familial PAH cases. Interestingly, RAGE has also been shown to interact with the BMPRII pathway in the pulmonary vasculature. The migratory effect of the BMPRII ligand BMP-2 in hPASMCs can be blocked by RAGE antagonism and likewise Mts1 induced-migration can be blocked with BMPRII short interference RNA showing cross talk between these pathways [12]. Thus increased activation of the Mts1/RAGE pathway may provide a 'second hit' risk factor in patients with a BMPRII mutation.

17β-estradiol (the predominant circulating estrogen in females) acts via RAGE to induce post-ischemic leukocyte adhesion in diabetic ovariectomized rats [13]. RAGE has also been hypothesised as important in determining the severity of breast cancer; the role of estrogens in the pathogenesis of breast cancer have previously been well described [14]. Interactions between estrogens and Mts1/RAGE remain to be investigated in the pulmonary circulation.

In this study, we investigated pulmonary vascular effects of female gender and 17β-estradiol on the Mts1/RAGE pathway *in vivo *and *in vitro *respectively. We show that female gender is permissive in the development of PAH in Mts1+ mice. Unlike their male counterparts, female Mts1+ mice develop plexiform-like lesions. Mts1 gene expression is increased in the lungs of female Mts1+ mice compared to male Mts1+ mice and this translates into greater expression of Mts1 protein in the distal pulmonary arteries. Physiological concentrations of 17β-estradiol increase Mts1 expression in hPASMCs, and sRAGE can inhibit 17β-estradiol-induced proliferation of these cells. This permissive effect of 17β-estradiol may contribute to the PAH phenotype observed in these female Mts1+ mice.

## Methods

### Animals

The investigation conforms with the United Kingdom Animal procedures act, 1986 and with the Guide for the Care and Use of Laboratory Animals published by the US National Institutes of Health (NIH publication No. 85-23, revised 1996).

### The S100A4/Mts1 mouse

Mts1+ mice were bred on a CBA x C57BL/6 background as described previously [15]. Mice were group-housed under standard laboratory conditions, with tap water and rat chow available *ad libitum*, on a 12 hr light/dark cycle. The PAH phenotype in transgenic mice is age-dependent and observed most clearly at 5 months of age. Accordingly, mice were studied at 5 months of age (25-35 g).

### Assessment of PAH indicators

#### In vivo hemodynamic measurements

Mice were anaesthetised using isofluorane supplemented with O_2 _(induction: 3%, maintenance: 1-1.5%). Pressure measurements were analysed as described previously [16]. Briefly, systemic arterial pressure was obtained via a microcannula (Harvard, 0.2 mm ID) inserted into a carotid artery. A 25-gauge needle was advanced into the right ventricle by use of a transdiaphragmatic approach for measurement of RVP. Data was recorded and analysed using a Biopac MP35 data acquisition system (Biopac Systems Inc, USA). Ten mice per group were studied.

#### Lung histology

One sagittal section was obtained from the left lung. Sections were stained with Elastica-Van Gieson stain and microscopically assessed in a blinded fashion for muscularisation of small pulmonary arteries (<80 μm external diameter) as described previously [16]. Lung sections from 4-6 mice from each group were studied. Approximately 150 arteries from each lung section were assessed.

#### Measurement of right ventricular hypertrophy

RVH was assessed by measuring the right ventricular free wall (RV) and left ventricle together with the septum (LV + S) separately. The ratio RV/LV+S was calculated.

### RNA Preparation and qRT-PCR

Total RNA was isolated using miRNeasy Mini Kit (Qiagen) and reverse-transcribed using Taqman reverse transcription kit (Applied Biosystems) according to the instructions of the manufacturer. Quantitative real-time PCR was performed using Universal master mix II with Assays on Demand gene expression probes (system and probes from Applied Biosystems) for S100A4/Mts1 (assay ID: Mm00803372_g1), RAGE (assay ID: Mm01134790_g1) and HMGCoA reductase (assay ID: Mm01282499_m1) using the comparative delta-CT method with β_2_-microglobulin as the endogenous control. GAPDH was used as a further endogenous control for HMGCoA reductase.

### Immunohistochemistry

Paraffin embedded lung sections (5 μm thick) were mounted on poly-L-lysine slides. Slides were dewaxed and sections rehydrated by immersion in ethanol (100%, 95%, and 70%) and then in distilled water. After antigen retrieval, endogenous peroxidase activity was blocked with 3% H_2_O_2 _in methanol for 30 minutes. Sections were pre-incubated in phosphate buffered saline supplemented with 1% bovine serum albumin, 10% normal horse serum for 1 hour. Endogenous biotin was blocked by use of an avidin/biotin blocking kit (Vector Laboratories) then incubated overnight with primary antibody (rabbit anti-α-smooth muscle actin 1:1000, Abcam; rabbit anti-von Willebrand factor 1:500, DakoCytomation; rabbit anti-Mts1 1:2000, supplied by Dr N. Ambartsumian; goat anti-rage 1:200, Abcam). Sections stained for α-smooth muscle actin and Mts1 were then exposed (1 hour) to biotin-labeled anti-rabbit secondary antibodies (Vector Laboratories) diluted 1:100, then to streptavidin-biotin- horseradish peroxidase solution. Sections stained for von willebrand factor and RAGE were exposed (1 hour) to horse radish peroxidase polymer secondary antibody (Vector Laboratories). RAGE staining was visualised using VIP peroxidise substrate kit (Vector) and appeared as red colouration. This provides optimum visualisation of RAGE staining in lung sections. Optimal visualisation of all other antibodies was acheived using 3',3'-diaminobenzidine tetrahydrochloride dihydrate and hydrogen peroxide. For Mts1 staining, nickel enhancement was used for optimizing contrast. Sections stained for α-smooth muscle actin, von Willebrand factor and Mts1 were counter-stained with haematoxylin. Eight mice per group were studied.

### Western blotting

Distal hPASMCs were supplied by Professor N. Morrell, Department of Medicine, University of Cambridge School of Clinical Medicine, UK [17]. hPASMCs were grown in high glucose Dulbecco's Modified Eagle Medium (Invitrogen, UK) containing 10% foetal bovine serum (Sera Laboratories International Ltd), and antibiotic, antimycotic solution (Sigma, UK). Cells were seeded out in 6-well plates, and when approximately 80% confluent, quiesced in 0.2% foetal bovine serum for 24 hours. Cells were then treated with 17β-estradiol (0.3-10 nM, Sigma, UK) for 4 hours, before being solubilised in detergent lysis buffer and electrophoresed using the NuPage system (Invitrogen, UK). Electrophoresed proteins were transferred onto a PVDF membrane (Millipore, UK), and blocked for an hour in 5% non-fat dried milk in Tris-buffered saline with 0.2% tween. Primary antibodies used were rabbit anti-Mts1 (1:1000) and mouse anti-alpha tubulin (1:5000; Abcam UK). Primary antibodies were applied overnight at 4°C, and blots thoroughly washed before addition of secondary antibody for one hour at room temperature (goat anti-rabbit IgG, 1:1500, or rabbit anti-mouse IgG 1:5000; all from Sigma, UK). All antibodies were diluted in 5% non-fat dried milk. The blots were thoroughly washed and then incubated with ECL reagent (Amersham Life Sciences, UK) and exposed to film. The molecular weight marker SeeBlue plus 2 (Invitrogen, UK) was used to calibrate the molecular weight range of the resolved proteins. After probing with the relevant primary antibody, the membrane was stripped and re-probed for the loading control alpha-tubulin. Densitometry was carried out using TotalLab software (Nonlinear Dynamics, UK). The density of the Mts1 protein band was expressed as a ratio of the density of the tubulin band. *n *= 6 experiments were performed.

### Proliferation of hPASMCs

hPASMCs were plated out in 24-well plates at a density of 20,000 cells/well. After 24 hours cells were quiesced in 0.2% foetal bovine serum for a further 24 hours. Cells were then exposed to 2.5% foetal bovine serum in addition to 17β-estradiol (1 nM) in the presence or absence of soluble RAGE (sRAGE, 2.5 μg/ml) for 5 days. sRAGE can bind RAGE agonists, thus diverting the agonist from the membrane bound form of the RAGE receptor and antagonising RAGE. sRAGE was added at least 30 minutes prior to addition of 17β-estradiol. Fresh media and drugs were added to the cells every 48 hours. Cell counts were performed using a haemocytometer, and counts from 4 experiments were repeated in duplicate.

### Statistical analysis

Inter-group statistical comparisons were made by one-way analysis of variance with Dunnetts post test or two-way analysis of variance with Bonferronis post test, as appropriate. Data are expressed as mean ± SEM.

## Results

### Effects of gender on pulmonary vascular remodeling in Mts1+ mice

**Figure 1:**
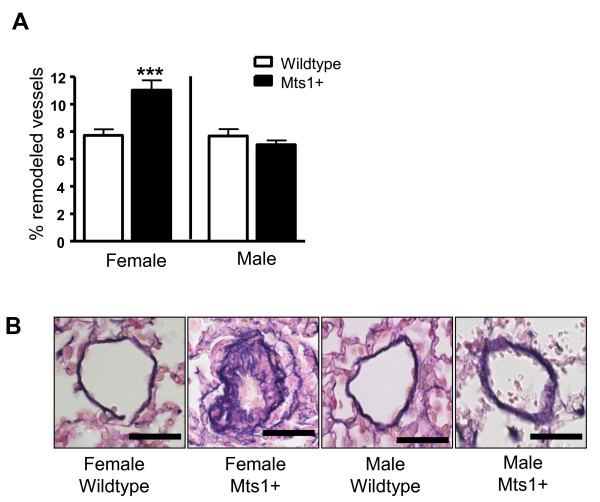
**The effects of gender on pulmonary vascular remodeling in Mts1+ mice**. **A**: Pulmonary vascular remodeling is increased in Mts1+ female mice compared to wildtype controls, but not in Mts1+ male mice (*n *= 4-6). Data are expressed as mean ± SEM and analysed by two-way analysis of variance followed by Bonferronis post-test. ***P < 0.001 cf female wildtype & male Mts1+ mice. **B**: Representative images (x400 magnification) of resistance pulmonary arteries (stained with Elastica Van Gieson) observed in female wildtype, female Mts1+ mice, male wildtype and male Mts1+ mice. Scale bars represent 50 μm.

**Figure 2:**
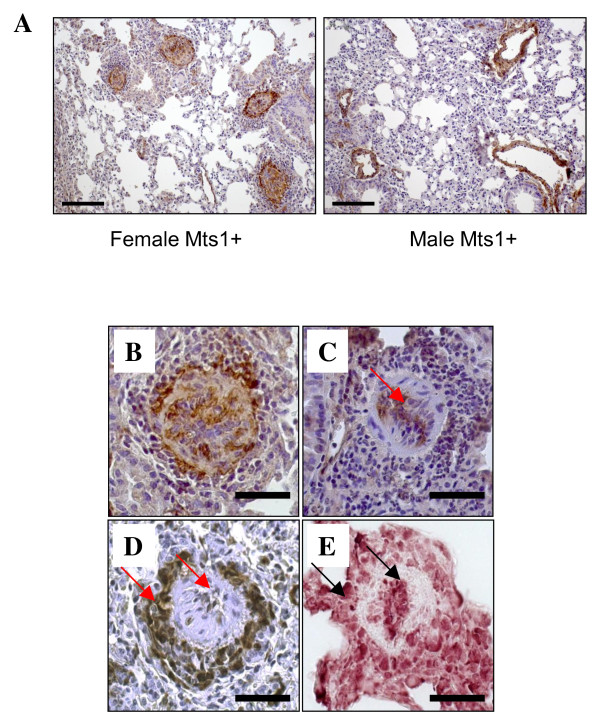
**Plexiform-like lesions occur in female Mts1+ mice**. A: Images of lung sections taken from female and male Mts1+ mice and stained with α-smooth muscle actin (x200 magnification). A cluster of plexiform-like lesions can be seen in the female Mts1+ mouse, whereas pulmonary arteries of a similar size in the male mouse show a much smaller smooth muscle cell layer and an intact lumen. Scale bars represent 100 μm. **B-E**: Representative images (x400 magnification) of plexiform-like lesions observed in female Mts1+ mice. Plexiform like lesions show extensive α-smooth muscle actin staining in the neointimal layer (B). Staining with von-Willebrand factor shows endothelial cells lining the obstructed lumen of the lesion (C). Mts1 (D) and RAGE (E) staining are evident in the endothelial and adventitial layers of the plexiform-like lesions Scale bars represent 50 μm.

Female Mts1+ mice exhibited an increase in pulmonary vascular remodeling compared to female wildtype mice. In contrast, male Mts1+ mice had no increase in pulmonary vascular remodeling when compared against their respective wildtype controls (Figure 1A&1B). In addition, we also observed that a subset of female Mts1+ mice (25%) exhibited severe remodeling in a small number of resistance arteries, which were characterized by neointimal formation and virtual lumen occlusion. The existence of these obliterative lesions was not observed in male Mts1+ mice (Figure 2A). Further examination of these lesions revealed that they were similar to lesions previously described by Greenway et al in a subset of Mts1+ mice (gender not documented) [6], and indicative of plexiform lesions observed in human PAH. Extensive α-smooth muscle actin staining was observed in the neointima of the plexiform-like lesions (Figure 2B). Endothelial cells lining the obstructed lumen of the pulmonary artery stained positive for von Willebrand factor (Figure 2C). Both Mts1 (Figure 2D) and RAGE (Figure 2E) staining was evident in the endothelial and advential layers of the plexiform-like lesions.

### Effects of gender on sRVP and RVH in Mts1+ mice

**Figure 3:**
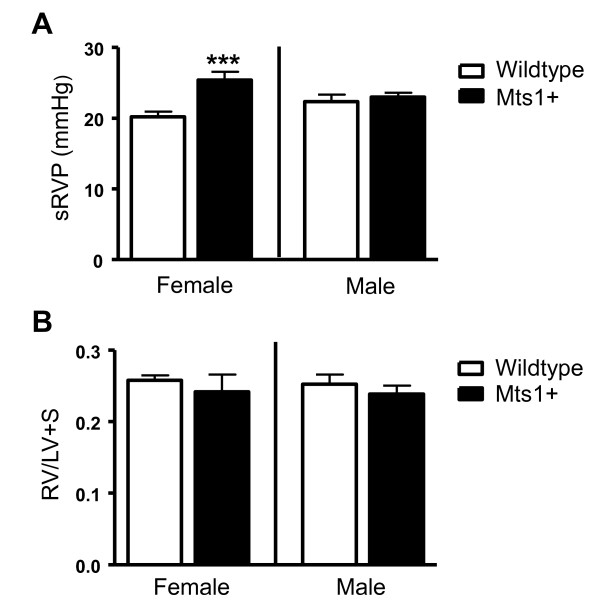
**The effects of gender on sRVP and RVH in Mts1+ mice**. **A**: sRVP is increased in Mts1+ female mice, but not in Mts1+ male mice (*n *= 5-9). **B**: RVH is unaffected in Mts1+ mice (*n *= 7-10). ***P < 0.001 cf wildtype controls. Data are expressed as mean ± SEM and analysed by two-way analysis of variance followed by Bonferronis post-test.

Female Mts1+ mice displayed an elevation of systolic right ventricular pressure (sRVP) compared with wildtype controls (Figure 3A). In contrast, male Mts1+ mice did not develop increased sRVP compared to their wildtype controls. Neither male nor female Mts1+ mice exhibited an increase in RVH compared to their respective wildtype controls (Figure 3B).

### Effects of gender on systemic arterial pressure in Mts1+ mice

The effects of over-expression of Mts1 and gender were selective for the pulmonary circulation, as neither mean systemic arterial pressure (mSAP) nor heart rate were affected (Table 1).

**Table 1:** The effects of gender and over-expression of Mts1 on mean systemic arterial pressure (mSAP) and heart rate (beats per minute, bpm) in mice

	mSAP (mmHg)	Heart Rate (bpm)
Wildtype female	99.5 ± 2.9 (6)	381 ± 11 (8)
Mts1+ female	92.7 ± 4.9 (8)	361 ± 10 (8)
Wildtype male	106.4 ± 4.9 (6)	383 ± 18 (6)
Mts1+ male	96.9 ± 5.4 (6)	384 ± 19 (6)

Data are expressed as mean ± SEM and analysed by two-way analysis of variance followed by Bonferronis post-test. *n *shown in brackets. No statistical significance was achieved.

### Effects of gender on expression of pulmonary Mts1 in Mts1+ Mice

**Figure 4:**
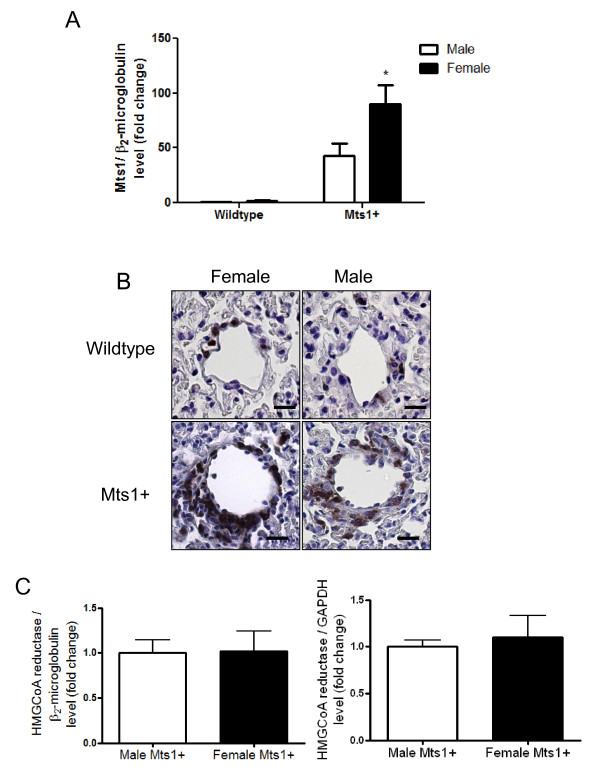
**Mts1 expression is increased female Mts1+ mice compared to males**. **A**: Mts1 gene expression is increased in the lungs of Mts1+ mice as assessed by qRT-PCR. This is exaggerated in lungs from female Mts1+ mice compared to male Mts1+ mice (*n *= 8). *P < 0.05 cf male Mts1+ mice **B**: Representative images of pulmonary arteries (x400 magnification) from female wildtype mice, male wildtype mice, female Mts1+ mice and male Mts1+ mice stained for Mts1. Mts1 expression is localised to the medial and adventitial layers of non-plexiform like pulmonary arteries and is most pronounced in female Mts1+ mice. Scale bars represent 20 μm. **C**: qRT-PCR analysis shows expression of the Mts1 promoter HMGCoA reductase is not influenced by gender in Mts1+ mice. Both β_2_-microglobulin and GAPDH were used as endogenous controls.

Having shown that Mts1+ female mice exhibit PAH whilst Mts1+ male mice remain unaffected, we wished to determine if differences in expression of Mts1 between male and female Mts1+ mice may contribute to this phenotype in females. Our qRT-PCR results show that, as expected, Mts1 mRNA was over-expressed in the lungs of Mts 1+ mice, however this was more pronounced in female mice than in males (Figure 4A). Mts 1 immuoreactivity appears localised to the medial and adventitial layers of the distal pulmonary arteries, with an exaggerated increase in Mts1 imminoreactivity in the medial layer of pulmonary arteries of female Mts1+ mice (Figure 4B). As Mts1+ mice were created using the HMGCoA reductase promoter we wished to rule out gender differences in the expression of this promoter in Mts1+ mice. We show equal expression of HMGCoA reductase between male and female Mts1+ mice using two housekeeping genes, β_2_- microglobulin and GAPDH (Figure 4C).

### Effects of gender on expression of pulmonary RAGE in Mts1+ Mice

**Figure 5:**
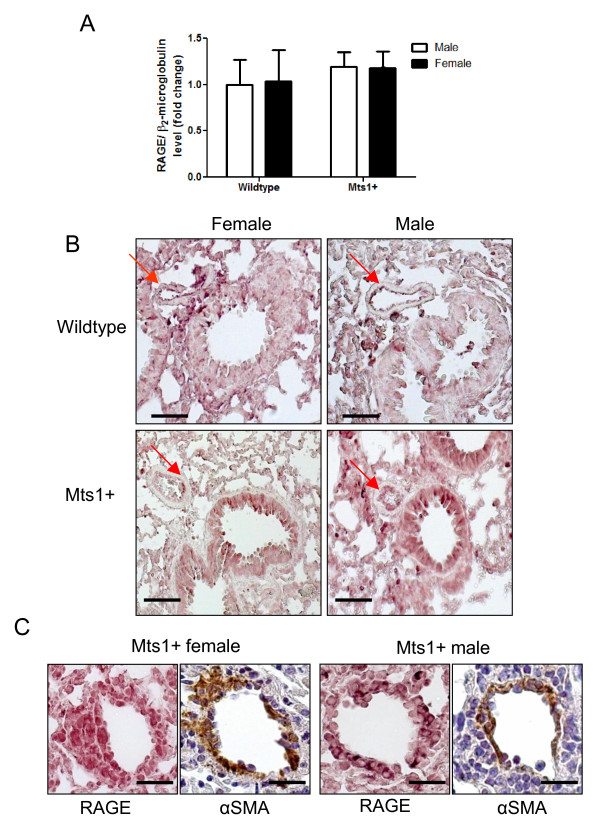
**Expression of RAGE is similar between male and female Mts1+ mice**. **A**: Whole lung RAGE gene expression is not affected by either over-expression of RAGE or gender as assessed by qRT-PCR. **B**: Representative images of whole lung sections (x200 magnification) from female wildtype mice, male wildtype mice, female Mts1+ mice and male Mts1+ mice stained for RAGE. RAGE immunoreactivity is localised to the endothelial layer of pulmonary arteries adjacent to airways and is enhanced in both male and female Mts1+ mice compared to wildtype controls. Red arrows point to pulmonary arteries. Scale bars represent 50 μm. **B**: Representative images of pulmonary arteries not adjacent to airways (x400 magnification) showing RAGE and α-smooth muscle actin immunoreactivity on adjacent lung sections in male and female Mts1+ mice. RAGE appears to be expressed in the medial and adventitial layers of pulmonary arteries not adjacent to airways. Scale bars represent 20 μm.

As Mts1 mediates its effects through interaction with the RAGE receptor, we wished to assess if RAGE expression also differed between male and female Mts1+ mice. Neither gender nor over-expression of Mts1 had any effect on the level of RAGE gene expression in the whole lung (Figure 5A). In line with this male and female Mts1+ mice had similar patterns of RAGE immunoreactivity in the distal pulmonary arteries (Figures 5B&5C). Although the data are not quantative, immunoreactivity for RAGE appeared heightened in lungs from both male and female Mts1+ mice compared to wildtype controls (Figure 5B). RAGE appeared localised to the endothelial cell layer of small resistance pulmonary arteries which were in proximity to airways (Figure 5B). We also observed RAGE staining in Mts1+ mice in pulmonary arteries not associated with airways. As the pattern of RAGE staining in these arteries was different to the endothelial staining in the airway-associated pulmonary arteries, we used α-smooth muscle actin immunohistochemistry in subsequent lung sections to localise RAGE staining. RAGE immunoreactivity appeared localised to both the medial and adventitial layers in non-airway associated pulmonary arteries from both male and female Mts1+ mice (Figure 5C).

### Interactions between 17β-estradiol and Mts1/RAGE in hPASMCs

**Figure 6:**
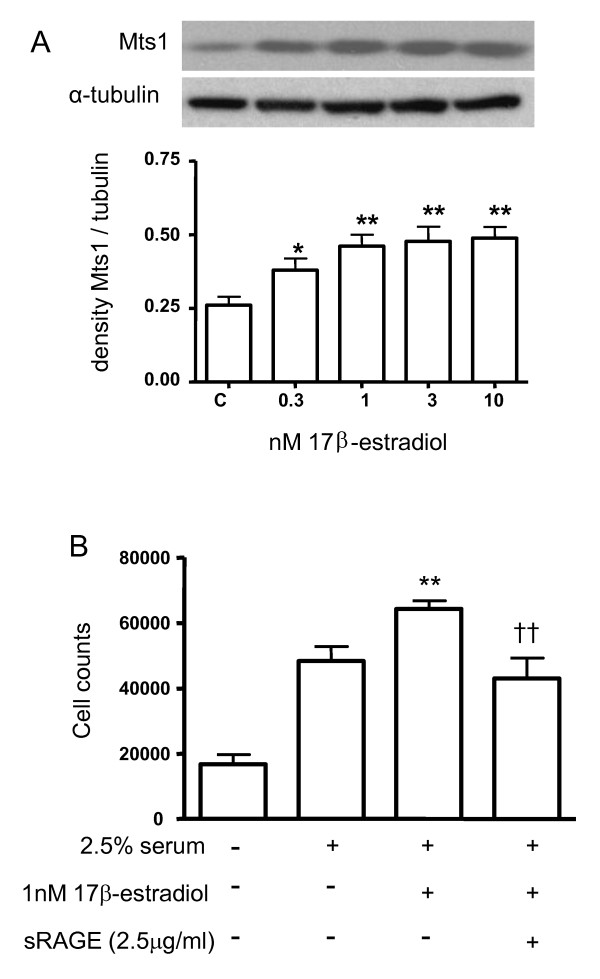
**Effects of 17β-estradiol on the Mts1/RAGE pathway in hPASMCs**. ****A****: 17β-estradiol (0.3-10 nM) induces an increase in Mts1 protein in hPASMCs. Quantification of data from multiple experiments (*n *= 4) is shown as mean ± SEM and analysed by one-way ANOVA followed by Dunnetts post-test. *P < 0.05, **P < 0.01 vs control. **B**: 17β-estradiol (1 nM) induced proliferation of hPASMCs is inhibited in the presence of sRAGE (2.5 μg/ml). Data are shown as mean ± SEM (*n *= 4) and analysed by one-way ANOVA followed by Dunnetts post-test. **P < 0.01 vs 2.5% serum, ††P < 0.01 vs 1 nM 17β-estradiol.

Having shown that female Mts1+ mice demonstrate increased Mts1 and RAGE expression, we wished to further investigate if this was likely mediated via 17β-estradiol as we have previously shown this to be implicit in the development of PAH in female mice overexpressing the serotonin transporter (SERT) [18]. To translate clinical relevance to our study, we adopted a human cell model to assess these 17β-estradiol effects. 17β-estradiol mediated a concentration-dependent increase in Mts1 expression in hPASMCs (Figure 6A). 17β-estradiol (1 nM) also stimulated proliferation of hPASMCs. This was inhibited by pre-incubation with the RAGE 'antagonist' soluble RAGE (sRAGE; 2.5 μg/ml; Figure 6B). Please note that it was not possible to generate sufficient amounts of sRAGE to examine the in vivo effects in our models.

## Discussion

We have presented evidence that the Mts1/RAGE pathway may play a role in the gender bias associated with PAH. Female Mts1+ mice develop increased pulmonary vascular remodeling and elevated right ventricular pressure compared to male Mts1+ mice. A subset of Mts1+ female mice (25%) also exhibit neointimal formation and lumen obliteration in a small number of pulmonary resistance arteries, consistent with the formation of plexiform-like lesions. These plexiform-like lesions are not apparent in male Mts1+ mice. Development of PAH and plexiform lesions have previously been described in Mts1+ mice, however in these studies there was no documentation of gender distribution and cohorts were likely mixed [6,19]. This, therefore, is the first study to show that the PAH phenotype in Mts1+ mice is specific to females. In accordance with Greenway et al. [6] we show that plexiform-like lesions are observed only in a sub-group of Mts1+ mice, and that these lesions are predominantly comprised of smooth muscle cells in the neo-intimal layer with a thin layer of endothelial cells lining the obliterated lumen. Also in agreement with Greenway et al, we show Mts1 expression to be in the endothelial and adventitial layers of the plexiform-like lesions. We further show that RAGE is also expressed in the endothelial and adventitial layers of the lesions in a similar pattern to Mts1. However, there are also some discrepancies between the current study and a previous study describing PAH in Mts1+ mice [19]. While we describe increased pulmonary vascular remodeling in female Mts1+ mice, the Mts1+ mice studied by Merklinger et al showed no pulmonary vascular remodeling [19]. In addition, we did not observe any differences in RVH in either male or female Mts1+ mice compared to controls. The original Mts1+ mice studied by Merklinger et al. showed increased RVH [19], but a later study by the same group showed Mts1+ mice to have similar RVH to control mice [20]. There are three notable differences between our study and that of Merklinger et al [19] which may contribute to the different results obtained. Our study used mice at 5 months of age whilst Merklinger et al. studied younger mice (~ 2months of age). In addition, our study has separated data from male and female mice whilst gender distribution was not described in the Merklinger et al. study and cohorts were likely mixed. Finally, Merklinger et al used C57BL6 mice as their wildtype controls while the current study employed C57BL6 x CBA mice as wildtype controls (Mts1+ mice are bred on a C57BL6 x CBA background) [15]. The reasons we studied mice at 5 months of age were two-fold. Firstly, we have demonstrated that the PAH phenotype does not present until 5 months of age in some transgenic mice, for example in mice over-expressing the serotonin transporter [21]. Secondly, female mice reach sexual maturity at 6-8 weeks of age, and can continue reproducing until around 9-12 months old, although litter sizes will drop around this time [22]. Thus at 5 months of age, female mice are mid-late reproductive age which approximates the age (average 35) in humans at which PAH presents itself.

We show female Mts1+ mice to develop increased sRVP despite a relatively small increase in pulmonary vascular remodeling. This is in line with previous reports describing increased sRVP in mice despite only 5-15% of vessels being remodeled [18,23]. Indeed, as discussed above, Merklinger et al showed increased sRVP and RVH in Mts1+ mice despite these mice having similar levels of pulmonary vascular remodeling to wildtype controls. Thus it is likely that factors additional to pulmonary vascular remodeling contribute to increased pulmonary pressures in mice. Indeed Mts1+ mice show decreased lumen diameter of the left pulmonary artery, and decreased attenuation of sRVP in response to nitric oxide, suggesting elevated pulmonary vascular resistance [7].

The dissociation between RVP and RVH observed in female Mts1+ mice is consistent with previous observations using female mice at this age. For example, dexfenfluramine-dosed female mice develop increased RVP in the absence of RVH, as do female mice over-expressing the human SERT gene [18,21,23]. We and others have shown that estrogen may have a protective effect against RVH [18,24], and this may explain the dissociation between RVH and RVP we observe in the present study.

Corresponding with the increased PAH phenotype observed in female Mts1+ mice, Mts1 gene expression was increased in the lungs of female Mts1+ mice compared to male Mts1+ mice. Immunohistochemical analysis showed increased Mts1 protein expression in the medial layer of small, resistance pulmonary arteries in female compared to male Mts1+ mice. These gender differences in Mts1 expression were not attributable to gender influences on the expression of the HMGCoA reductase promoter. Thus our results suggest an influence of sex hormones on Mts1 expression and subsequent PAH phenotype and we wished to assess the effects of 17β-estradiol on Mts1 expression. To determine if our in vivo results in mice could be translated to a human cell model, we chose to study hPASMCs. In line with female Mts1+ mice showing increased expression of Mts1 in the medial layer of pulmonary arteries, physiologically relevant concentrations of 17β-estradiol up-regulated Mts1 expression in hPASMCs. Mts1 has previously been shown to act in an autocrine fashion in hPASMCs; it is released from and then acts upon these cells to mediate proliferation and migration [10]. Thus increased expression of Mts1 within the medial layer of small resistance pulmonary arteries may contribute to the increased remodeling observed in female Mts1+ mice. Mts1 exerts its effects on proliferation and migration in hPASMCs via RAGE [10]. Interestingly, we show sRAGE to inhibit 17β-estradiol-induced proliferation of hPASMCs. As Mts1 is the ligand for RAGE, collectively, the results suggest that 17β-estradiol induces increased expression of Mts1 that then activates RAGE and stimulates hPASMC proliferation.

The mechanism by which Mts1 is released from hPASMCs by serotonin involves co-operation between the SERT and the 5-HT_1B _receptor [10]. In line with this, Mts1 mRNA is up-regulated in the lungs of female mice over-expressing SERT (SERT+ mice) [10]. We have recently shown that female gender is permissive in the development of PAH in SERT+ mice [18]. In addition we have shown a critical role for 17β-estradiol in the development of PAH in female SERT+ mice as ovariectomy reduces the PAH phenotype which can then be re-established using subcutaneous 17β-estradiol implants [18].

Previous studies have demonstrated that female gender and/or estrogens can be protective in experimental models of PAH, such as the hypoxic and monocrotaline models and mice lacking the vasoactive intestinal peptide gene [4,5,25]. This suggests that, in certain circumstances, female sex hormones may actually be protective against PAH. Indeed, the estradiol metabolite 2-methoxyestradiol (2-ME) has been shown to mediate protective effects in monocrotaline induced PAH [26]. Estradiol is metabolized to 2-hydroxyestradiol (2-OHE) mainly via the estrogen metabolizing enzymes CYP1A1/2 and to a lesser extent via CYP1B1. 2-OHE is then converted to 2-ME via catechol O-methyltransferase. Estradiol can also be metabolized to 16α-hydroxyestrone (16-OHE1) via various CYP enzymes including CYPs 1A1, 1A2 and 1B1 [27]. 2-OHE/2-ME may have anti-proliferative effects on cells [26], while 16α-OHE1 stimulates cellular proliferation by constitutively activating the estrogen receptor [28]. Hence, both pro- and anti-proliferative effects of estrogens may be observed, depending on their metabolism. Consistent with this, both gene and protein expression of CYP1B1 is increased in PASMCs from PAH patients [29]. In addition, higher penetrance of PAH is observed among BMPRII mutation carriers with a polymorphism in the CYP1B1 genotype [30]. Interestingly, the 2-OHE/16α-OHE1 ratio was also decreased in PAH patients with a BMPRII mutation compared to unaffected BMPRII mutation carriers [30]. Disruption in the balance of estrogen metabolites may therefore account for the differential effects of female sex hormones in different models of PAH.

## Conclusions

We have identified that female gender is permissive in the development of PAH in 5 month old Mts1+ mice. This may be related to increased Mts1 expression in these females. 17β-estradiol can up-regulate Mts1 expression and 17β-estradiol-induced proliferation of hPASMCs is dependent upon RAGE activation. Therefore the 17β-estradiol/Mts1/RAGE axis may play a role in the development of PAH, and contribute to the gender bias associated with this disease.

## Abbreviations

BMPRII: bone morphogenetic protein receptor type 2; hPASMCs: human pulmonary artery smooth muscle cells; Mts1: S100A4/Mts1; Mts1+ mice: mice over-expressing Mts1; PAH: pulmonary arterial hypertension; RAGE: receptor for advanced glycosylation end products; RVH: right ventricular hypertrophy; SERT: serotonin transporter; SERT+ mice: mice over-expressing the serotonin transporter; sRAGE: soluble receptor for advanced glycosylation end products; sRVP: systolic right ventricular pressure

## Competing interests

The authors declare that they have no competing interests.

## Authors' contributions

YD participated in the design of the study, carried out the in vivo and cellular studies, participated in the immunohistochemistry, performed the statistical analysis and drafted the manuscript. MN carried out the immunohistochemistry. KW participated in the design of the study and the immunohistochemistry. KM participated in the design of the study and the immunohistochemistry. LL genotyped the mice. NA synthesized the Mts1 antibody, and provided the Mts1+ mice. MR participated in the study design and drafted the manuscript. MRM acquired funding for the study, participated in its design and co-ordination and drafted the manuscript. All authors read and approved the final manuscript.
